# Association of exercise capacity and endothelial function in patients with severe exacerbations of chronic obstructive pulmonary disease

**DOI:** 10.1038/s41598-020-80601-w

**Published:** 2021-01-11

**Authors:** Erika Zavaglia Kabbach, Alessandro Domingues Heubel, Cassia da Luz Goulart, Valéria Amorim Pires Di Lorenzo, Shane A. Phillips, Audrey Borghi-Silva, Renata Gonçalves Mendes

**Affiliations:** 1 Department of Physical Therapy, Federal University of Sao Carlos, Road Washington Luís, Km 235, Jardim Guanabara, Sao Carlos, Sao Paulo, 13565-905 Brazil; 2 Department of Physical Therapy, College of Applied Health Sciences, University of Illinois at Chicago, Chicago, USA

**Keywords:** Cardiovascular diseases, Respiratory tract diseases, Diseases, Respiratory signs and symptoms

## Abstract

Severe acute exacerbations of chronic obstructive pulmonary disease (AECOPD) are associated with significant poor outcomes including an increased risk of cardiovascular (CV) events and exercise intolerance. Endothelial dysfunction might contribute to an impaired vascular homeostasis and consequently to CV events and exercise capacity. This study aimed to evaluate the association between exercise capacity and endothelial function in patients with severe AECOPD. Forty-five COPD patients diagnosed with severe AECOPD and admitted to the University Hospital of São Carlos from 2017 to 2019 were enrolled in this observational clinical study. Endothelial Function was assessed by brachial artery ultrasonography (M-Turbo, Sonosite, Bottle, WA, USA) and Flow Mediated Dilatation (FMD) technique in absolute (mm) and percentage values (%). Walking distance (6MWD) obtained by six-minute walk test was considered to characterize the exercise capacity. Pearson’s correlation analysis and linear regression model were applied and a significance level of 5%. There was a significant positive correlation between exercise capacity and endothelial function. Pearson correlation coefficient were 0.36 (p = 0.02) and 0.40 (p = 0.01) between 6MWD and FMD in mm and %, respectively. Linear regression model revealed 6MWD (p = 0.007), accounting for 15% of FMD (%) variance (R^2^ adjusted). FMD (%) = 2.11 + (0.0081*6MWD). Exercise capacity is associated with endothelial function in patients with severe AECOPD. FMD was found to be increasing with increasing walked distance. Further research is needed to provide evidence of effectiveness of rehabilitation on exercise capacity and endothelial function in these patients and its prognostic value.

## Introduction

Chronic Obstructive Pulmonary Disease (COPD) is the third leading cause of death worldwide, affecting many people in low-income, middle-income, and wealthy countries^1,2^. On the course of the disease, many patients experience an acute exacerbation of COPD (AECOPD), characterized as an acute worsening of respiratory symptoms, which may require additional therapy^3,4^. Acute exacerbation also negatively affects patients’ health status^5^, exercise capacity^6^, lung function^7^ with a detrimental and prolonged impact in outcomes.

Severely distressing events requiring hospitalization impact greatly on physical activity capacity and functional state, which are markers of increased future risk^8^. Interestingly, a multicenter study involving more than 16,000 patients found that AECOPD also contributes to an increased risk of subsequent cardiovascular (CV) events (myocardial infarction, unstable angina and transient ischemic attack), with a tenfold increased hazard for CV events in those hospitalized with severe AECOPD^9^.

Some of the literature supports AECOPD as a trigger for CV events^10–12^. An more activated local and systemic inflammation, increased levels of fibrinogen and interleukin‐6, and hypoxemia resulting in a prothrombotic environment, higher arterial pressures, arterial stiffness and endothelial dysfunction may predispose to CV events during AECOPD^11,12^. In order to reduce the burden of COPD and given the acceptance of the prognostic significance of CV in AECOPD^10^, a heightened comprehensive investigation and an integrated approach for better management of this population at high risk is imperative.

As mentioned, dysfunctional endothelium is a well-established hallmark feature of CV disease^13,14^. Increased reactive oxygen species and inflammation, diminished nitric oxide (NO) production and bioavailability are the main mechanisms underlying the pathophysiology of endothelial dysfunction^13^. Unhealthy vascular endothelium is related to systemic inflammation and atherosclerosis resulting in higher cardiovascular risk in COPD^15^. Endothelial dysfunction has been widely recognized in stable and exacerbated COPD patients^16–19^.

Furthermore, endothelial dysfunction might contribute to dysregulation of muscle proteins balance and inadequate muscle perfusion, which may be related to a pathophysiological mechanism linking reduced exercise capacity, muscle weakness and CV disease in AECOPD^20,21^. Ideal blood flow to peripheral muscles is also endothelium-dependent and is crucial for normal perfusion of working muscle during activities^22^. Undeniably, exercise-induced increases in blood flow and shear stress are physiological stimuli for enhanced production and activity of NO, which has a key role in healthy functioning of the endothelium^23^.

In stable COPD patients, exercise capacity assessed by six-minute walking distance was independently related to endothelial dysfunction^24^. In this sense, the maintenance of functional capacity may play a role in preserving endothelial function in stable COPD patients^24^. Although severe acute exacerbation of COPD has a subsequent increase in CV risk and poor functional outcomes^24^, the relationship between endothelial function and exercise properties has not been investigated.

The current study aimed to evaluate the association between exercise capacity and endothelial function in patients with severe AECOPD. We hypothesized that exercise capacity would be positively associated with endothelial function in AECOPD patients. This study may provide a valuable knowledge for future clinical studies focusing the effect of rehabilitative strategies to enhance exercise capacity on endothelial function, CV risk and outcomes during AECOPD.

## Methods

### Ethics aspects

All methods were carried out in accordance with relevant guidelines and regulations (Declaration of Helsinki). The study was approved by the Research Ethics Committee of the Federal University of São Carlos (Brazil, reference number 46431415.0.0000.5504) and University Hospital of São Carlos. All the subjects and/or responsible individual were informed about the study objectives, experimental procedures and insured the confidentiality of data collected. All subjects gave written informed consent before the study’s initiation.

### Subjects

Forty-five COPD patients diagnosed with severe AECOPD and admitted to the University Hospital of São Carlos (HU-UFSCar) from 2017 to 2019 were enrolled in this observational clinical study. To confirm COPD diagnosis and stratification, all patients attended the laboratory pulmonary function testing (spirometry), thirty days after hospital discharge (stable condition). COPD diagnosis was confirmed with post-bronchodilator spirometry—FEV_1_/forced vital capacity ≤ 0.7 and post-bronchodilator FEV_1_, ≤ 80% predicted in stages I, II, III, or IV)^25^. AECOPD was clinically defined as an acute worsening of respiratory symptoms that results in additional therapy^26^ and classified as severe according GOLD as patients requiring hospitalization^25^.

Assessments during AECOPD occurred during hospitalization at least 24 h and within 48 h after starting standard therapy for AECOPD (beta-2 agonist, anticholinergics, oral corticosteroids, oxygen therapy and antibiotic treatment)^25^ at the University Hospital of São Carlos and then 30 days following AECOPD at Cardiopulmonary Laboratory of Federal University of São Carlos.

The exclusion criteria were: peripheral vascular disease, neurological conditions that would preclude participation in the required protocol, other concomitant respiratory diseases, mechanical ventilation, hemodynamic instability, unstable angina or myocardial infarction history in the last 6 months and patients over 80 years.

### Protocol

1. During hospitalization: clinical characterization, endothelial function, 6 min Walking Distance (6MWD), modified Medical Research Council (mMRC) dyspnea scale and COPD Assessment Test (CAT) were assessed. Arterial blood gas and serum C-reactive protein (CRP) concentrations were measured at rest with ambient air or with a O_2_ catheter when the patient already used it.
2. After 30 days: lung function test (spirometry) was performed according to the American Thoracic Society/European Respiratory Society guidelines^27^.

#### Endothelial function

**Figure 1:**
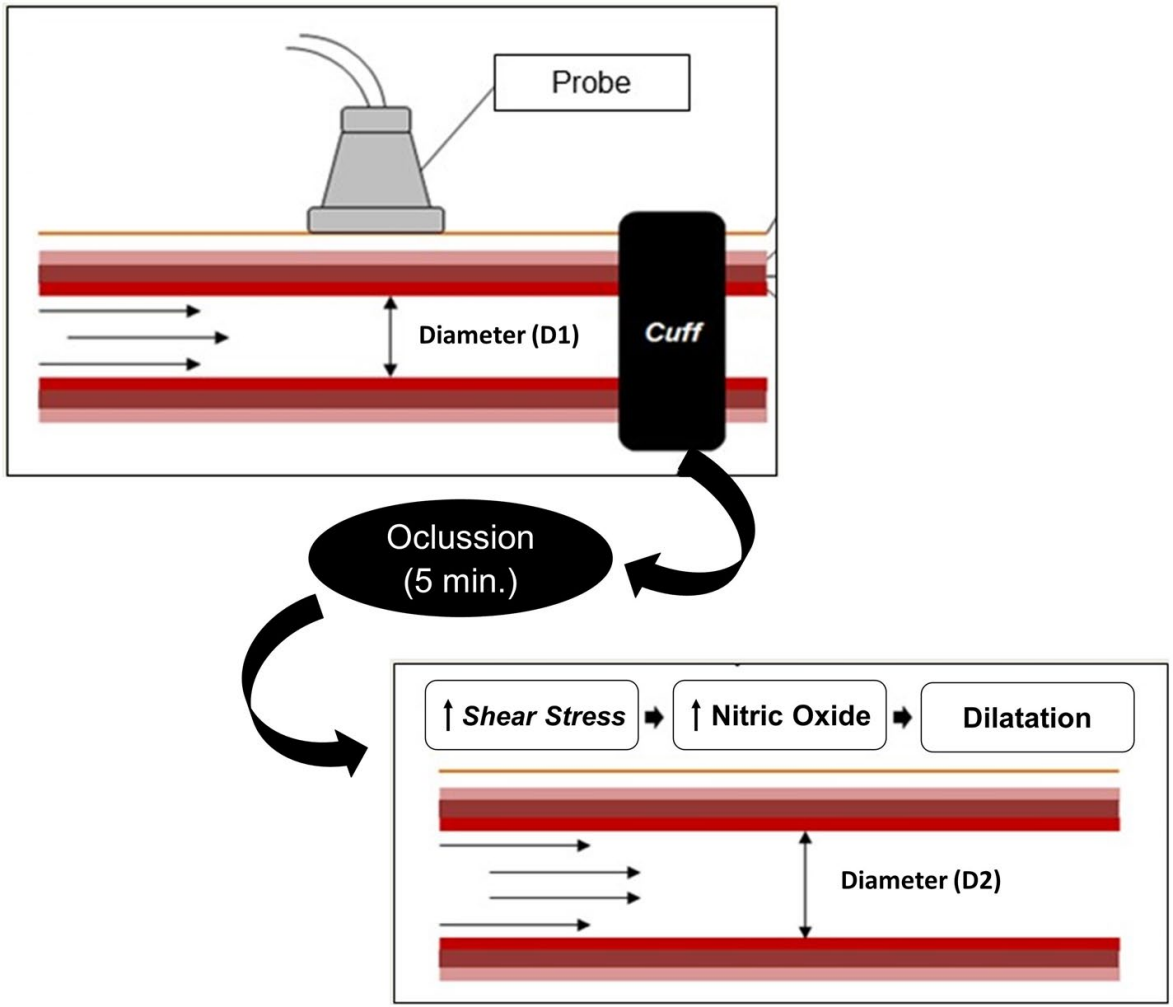
Measurement of FMD. The baseline diameter (D1) between the proximal and distal intima was recorded before RH. The *Cuff* was inflated at 200 mmHg during 5 min. Immediately after brachial artery ischemia, the diameter post RH (D2) was recorded. The release of cuff should cause increased flow to the region, promoting increased shear stress, greater release of nitric oxide and, consequently, vasodilation. FMD was expressed as the change in percentage: [(baseline diameter − diameter post RH)/baseline diameter × 100] and absolute (diameter post RH − baseline diameter).

Endothelial function was assessed noninvasively by Flow Mediated Dilatation (FMD) of the brachial artery using ultrasonography (M-Turbo, Sonosite, Bottle, WA, EUA) based on endothelium-dependent vasomotor reactivity and according described previously^28,29^. Longitudinal images of the right brachial artery were obtained with a high-frequency probe (10 MHz) proximal to the antecubital fossa and the diameter and central flow velocity (pulsed Doppler) were measured. Reactive hyperemia (RH) was induced by the inflation of a cuff positioned around the forearm at 200 mmHg during 5 min. To assess FMD, peak and mean blood flow velocity they were measured within the first 10 s after cuff release, and the diameter was continuously recorded (at a rate of 7.5 images/sec) for 3 min (Fig. 1). Digitally recorded images were later analyzed using the software: Brachial Imager (Medical Imaging, Iowa City, IA, USA). FMD was expressed as the change in percentage: [(baseline diameter − diameter post RH)/ baseline diameter × 100] and absolute values (diameter post RH—baseline diameter)^28,29^. To perform an estimation of the shear stress (shear rate—SR) the following calculation was performed: SR = 8 × mean blood velocity (cm/s^−1^)/internal vessel diameter (mm)^28^. The normalized FMD by shear-rate was calculated using the following formula: FMD (%)/SR (s)^30^.

#### Six-minute walk test (6MWT)

The 6MWT was performed following American Thoracic Society/European Respiratory Society guidelines to evaluate exercise capacity^31^. Subjects were asked to walk as far as possible along a 30-m corridor in 6 min. The following measurements were taken at the beginning, during (second and fourth minute) and at the end of the test: blood peripheral oxygen saturation (SpO_2_) using an oximeter, heart rate (HR) using a heart rate monitor (Polar, Oulu, Finland), and dyspnea sensation. Blood pressure (BP) was measured at the beginning and at the end of testing with a sphygmomanometer (Welch Allyn1, Skaneateles Falls, New York, USA) and stethoscope (Littmann, Saint Paul, Minnesota, USA). Patients with hypoxemia or who presented with SpO_2_ < 85% during the test were supplemented with oxygen. Physical therapist walked beside the patient pulling the portable cylinder trolley. The walking distance in meters was used for the analyses. Predicted values were calculated according to reference for Brazilian population: 6MWD_pred_ = 890.46 − (6.11 × age) + (0.0345 × age^2^) + (48.87 × gender) − (4.87 × BMI)^32^.

### Statistical analysis

The Shapiro–Wilk test was used to verify the normality of data. Clinical and endothelial function data, continuous variables, were expressed as mean (± standard deviation); categorical variables were quantified as numbers and percentages compared to the entire population. Pearson’s correlations coefficients were used to test the association between 6MWD and FMD. The magnitude of correlations was determined considering the following classification scheme for r-values: low (0–0.25), moderate (> 0.25–0.50), strong (> 0.50–0.75), and very strong (> 0.75). Linear regression model was generated to determine the association of the exercise capacity (6MWD), as an independent variable on dependent variable of vascular function (FMD%). All the assumptions (independence of values, linearity of the means, data normality and homoscedasticity in variance values) of the linear regression model were verified. Statistical analysis were performed using SigmaPlot Version 11.0 (SyStat Software, Inc., San Jose, CA) and the accepted level of significance was p ≤ 0.05. Sample size was previously calculated using G*Power 3.1, in order to predict exercise capacity using FMD (%), considering a statistical power of 80%, assuming a medium effect size of 0.2, with an alpha value at 0.05.

## Results

**Figure 2:**
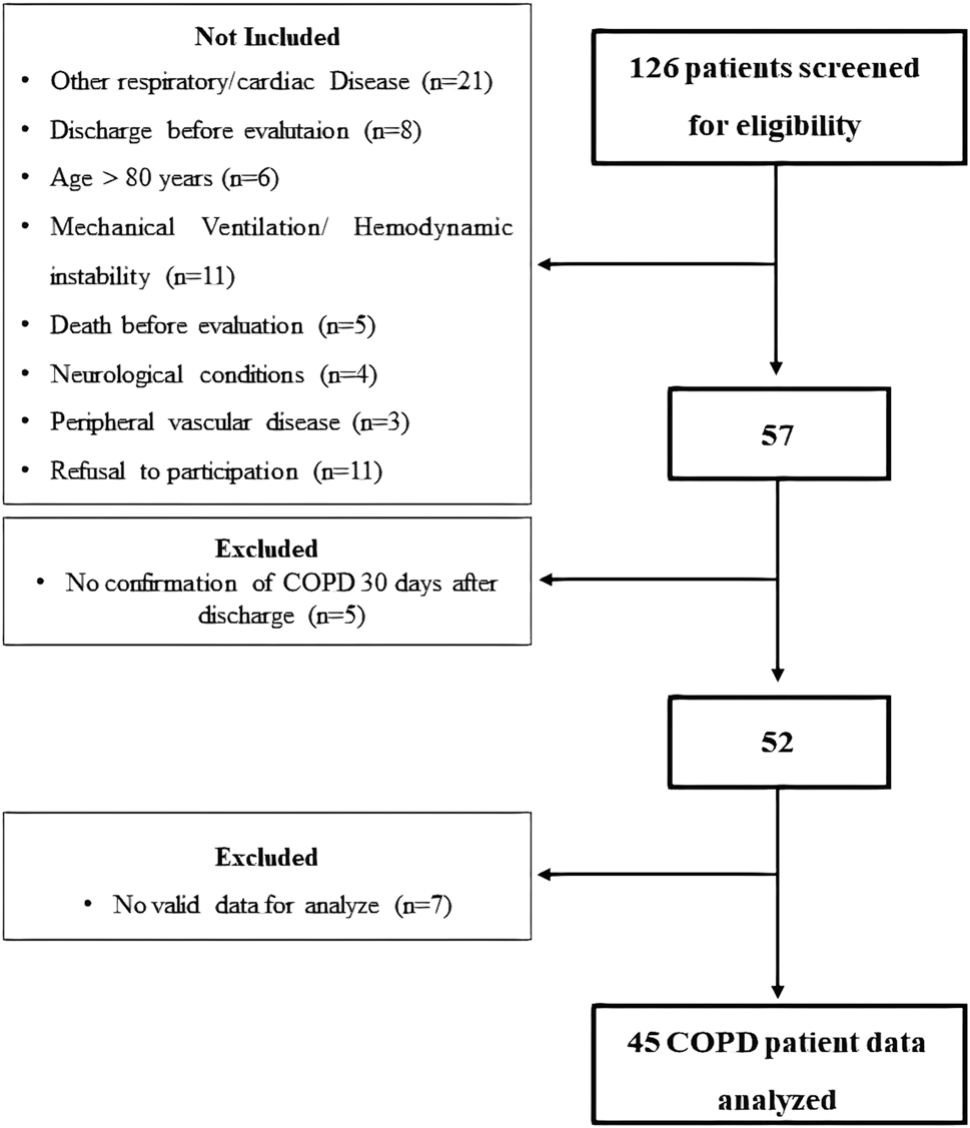
Study flow. *COPD* chronic obstructive pulmonary disease;

One hundred and twenty-six were initially recruited for this study, forty-five (68.2 ± 8.0 years) patients completed the study and were included for the analysis (Fig. 2). The clinical data and physiological parameters, lung function, main comorbidities and medications of the patients are shown in Table 1. A gender balance, GOLD classification of airway limitation severity staged II-IV and reduced exercise capacity (6MWD, predicted %) were observed.

**Table 1:** Baseline patients’ features.

Clinical data	n = 45
Age, years	68.2 ± 8.0
Male, n (%)	23 (51)
BMI, kg/m^2^	24.7 ± 5.5
Exacerbation, n/ per year	1.9 ± 1.3
Smoking, never/ex/current	1(3%)/29(64%)/15(33%)
Pack-years	60.0 ± 51.5
HR, bpm	85.8 ± 18.9
RR, rpm	21.7 ± 3.4
SBP, mmHg	125.6 ± 19.6
DBP, mmHg	76.2 ± 10.6
SpO_2_, %	92.4 ± 3.3
PaO_2_, mmHg	70.5 ± 24.7
PaCO_2_, mmHg	44.7 ± 11.6
CRP, mg/dl	6.1 ± 6.0
Supplemental O_2_, n (%)	35 (78)
mMrC	3 (2–3)
CAT	23.7 ± 8.7
6MWD, meters	230.5 ± 107.5
6MWD, predicted (%)	38.7 ± 18.2
**Main comorbidities, n (%)**	
Systemic arterial hypertension	24 (53)
Type 2 diabetes	8 (18)
Chronic stroke	5 (11)
Previous myocardium infarction	6 (13)
**Lung function**	
Forced vital capacity, % predicted	71.9 ± 18.8
FEV_1_/forced vital capacity	0.56 ± 0.19
FEV_1_, % predicted	48.5 ± 16.2
COPD Gold Stage II, n (%)	13 (29)
COPD Gold Stage III, n (%)	25 (55)
COPD Gold Stage IV, n (%)	7 (16)
**Medications, n (%)**	
Antibiotic therapy	41 (91)
Short-acting beta agonist (saBa)	28 (62)
Long-acting beta agonist (laBa)	4 (9)
Anticholinergic (saMa and laMa)	37 (82)
Systemic corticosteroids (SCs)	38 (84)
Systemic arterial hypertension	26 (58)
Others	37 (82)

Data are presented as mean ± SD or median (interquartile).

*BMI* body mass index; *HR* heart rate; *RR* respiratory rate; *SBP* systolic brachial pressure; *DBP* Diastolic Brachial Pressure; *sPO*_*2*_ oxygen saturation; *PaO*_*2*_ partial pressure of oxygen; *PaCO*_*2*_ partial pressure of carbon dioxide; *CRP* C-reactive protein; *mMrC* modified Medical Research Council; *CAT* COPD Assessment Test; *6MWD* six minute walking distance; *FEV*_*1*_ forced expiratory volume in first second; *laBa* long-acting Beta2-Agonist; *SCs* systemic corticosteroids; *laMa* long-acting anticholinergics; *saBa* short-acting Beta2-agonist; *saMa* Short-acting anticholinergics.

Endothelial function values at AECOPD are demonstrated in Table 2.

**Table 2:** Endothelial function parameters by brachial artery measurement.

	AECOPD patients, n = 45
Baseline diameter, mm	4.51 ± 0.63
FMD, mm	0.19 ± 0.10
FMD, %	4.20 ± 2.12
Baseline velocity, cm/sec	19.64 ± 9.49
RH flow velocity, cm/sec	37.56 ± 16.55
RH *Shear-rate*, s	66.76 ± 35.06

Data are presented as mean ± SD.

*AECOPD* acute exacerbation of chronic obstructive pulmonary disease; *mm* millimeter; *FMD* flow-mediated dilatation; *RH* reactive hyperemia; *%* relative change.

**Figure 3:**
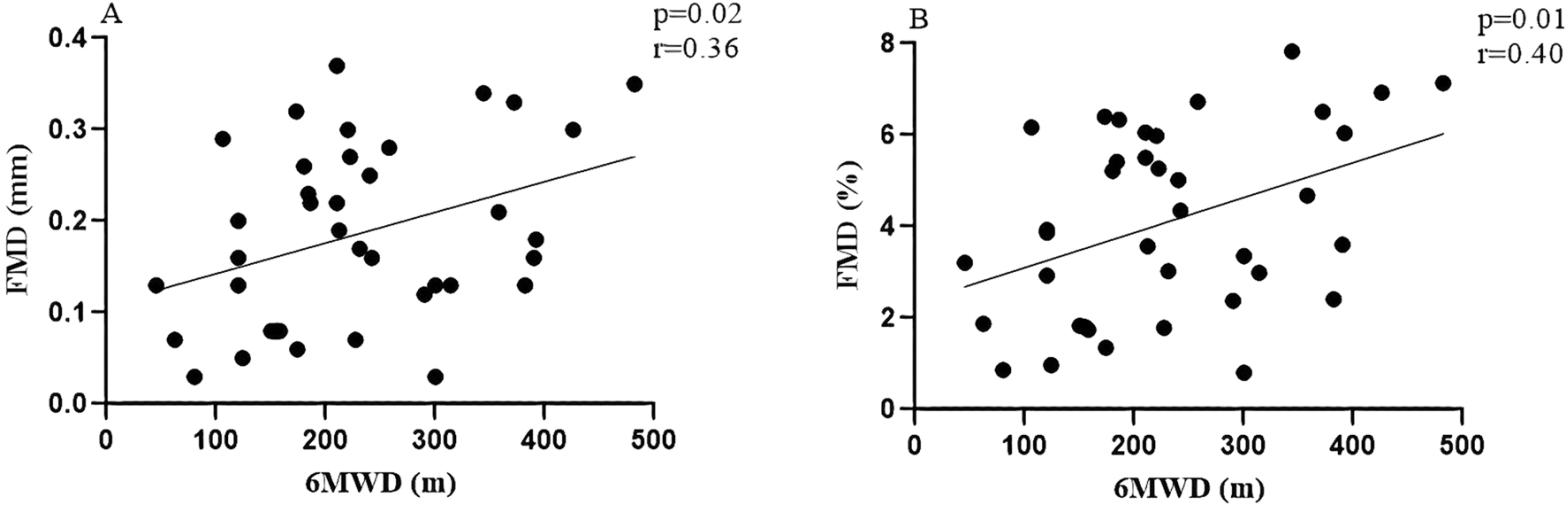
Correlations between exercise capacity and endothelial function. Data are presented as the correlation coefficient (r) and p <  0.05. Relationship between exercise capacity and endothelial function: (**A**) The FMD (mm) was positively associated with the 6MWD; (**B**) The FMD (%) was positively associated with the 6MWD. FMD (mm): absolute values; 6MWD was expressed in meters. The figure was create using SigmaPlot version 11.0, from Systat Software, Inc., San Jose California USA, www.systatsoftware.com.

Statistically significant associations were found between exercise capacity and endothelial function in severe acute exacerbation of COPD (Fig. 3). Pearson correlation test showed coefficients of 0.36 (p = 0.02) and 0.40 (p = 0.01) between 6MWD and FMD in mm and %, respectively. Linear regression analysis resulted in a significant correlation between 6MWD and endothelial function (%), p = 0.007, ß = 0.0081, t = 2.83, accounting for 15% of the variance (adjusted R^2^). The simple linear regression model was FMD (%) = 2.11 + (0.0081*6MWD) (Table 3). The regression coefficient associated with 6MWD demonstrated that each one-unit increase in walking distance is associated with a 0.0081 unit increase in FMD.

**Table 3:** Linear regression model between exercise capacity and FMD (%).

Variables	β coefficient	Std. Error	*p*-value
Constant	2.11	0.747	0.007
6MWD (m)	0.0081	0.0028	0.007

Adjusted R^2^ = 0.15; F = 8.04 (p = 0.007).

## Discussion

This study aimed to evaluate the association between exercise capacity and endothelial function in patients with severe AECOPD. The present study findings revealed that FMD was found to be increasing with increasing walked distance. Exercise capacity was able to explain 15% of the FMD variance, which is a recognized causative factor of cardiovascular disease during exacerbation period.

### Exercise capacity and endothelial function in AECOPD

In COPD patients, 6WMD has been considered a potentially useful biomarker of disease severity, evaluating pulmonary, cardiovascular and muscular systems integrated responses^33,34^. The 6MWD below 350 m has been used to identify subsets of the COPD population at higher risk of exacerbation-related admission or death^35^. As previously reported, AECOPD is marked by important functional injuries. Bed rest during hospitalization and reduced physical activity, poses a potent threat to muscles and consequently to functional capacity. In agreement with the literature, we found a reduced distance covered of 38% of the predicted value (230 m) and 43% (268 m) was found in the study of Pitta et al.^8^. Another previous study, aimed to evaluate effects of aerobic exercise also find a distance of 224 m 48 h after exacerbation in patients during hospitalization for exacerbation of COPD^36^. Therefore, exercise capacity deserves attention at this moment independently, however with promising association with vascular health.

A properly functioning endothelium normally plays a crucial role in providing appropriate hemostatic balance. Nonetheless, a dysfunctional endothelium is seen during exacerbation related mainly to high inflammation and oxidative stress imbalance^37^. This abnormal condition is characterized by reduced vasodilation ability, and both pro-inflammatory and pro-thrombic states, creating favorable conditions for platelet, leukocyte and cytokines activation and adhesion towards damage of the arterial wall^38,39^ which can favor CV events. In this study, we used the FMD method, which is a widely accepted evaluation method of the vasodilator function of the endothelium by stimulating reactive hyperemia.

To our knowledge, no previous study investigated the association between endothelial function and exercise capacity status during severe AECOPD. Minet et al.^24^ found that the 6MWD was independently associated with reactive hyperemia by peripheral arterial tonometry (RH-PAT) ($\beta :$ 0.00768; SE: 0.00249; p = 0.0040) suggesting that an impaired functional capacity may be a main predictor of endothelial dysfunction in COPD patients^24^. Clarenbach et al. showed that patients with severe airflow obstruction who were physically active were less prone to more severe impairments of FMD, compared to inactive COPD patients with severe airflow obstruction, suggesting that physical activity may attenuate the progression of vascular dysfunction in COPD^40^. Vaes et al.^41^ also demonstrated a significant association between reduced maximal aerobic capacity (VO_2_ peak) and impaired peripheral endothelial function in patients with stable COPD.

In this way, our findings revealed significant associations between exercise capacity and endothelial function in severe acute exacerbation of COPD. FMD was found to be increasing with increasing walked distance. The coefficient of determination observed was of 0.15, which means that 15% of the variance in FMD is due to exercise capacity in patients with COPD and AECOPD. The remaining 85% could be due to individual variation and might be explained by factors that were not taken into account in the analysis, such speculative factors as previous comorbities and endothelial function healthy, previous exercise and rehabilitation effects, inflammation profile and medications.

Regarding shear stress, it is well accepted as an important stimulus for the vascular dilation reactivity response^42^. Shear stress is able to stimulate a quiescent endothelial cell phenotype that is anti-inflammatory, vasodilatory, antithrombotic and pro-atherogenic^42^. In this context, exercise-induced increases in blood flow and shear stress have been observed to enhance vascular function. Increased blood flow enhances endothelium-dependent vasodilatation by increased expression of endothelial NO synthase and the release of NO and prostacyclin^43^. NO and prostacyclin inhibit multiple processes involved in atherogenesis. In addition, increase flow modulates the expression of a panoply of paracrine substances, including endothelial growth factors^43^. All of these processes may contribute to the beneficial effects of exercise-induced vascular remodeling and reactivity^43^.

Thus, it may be expected that patients who have a better capacity for exercise during hospitalization, may present with some benefits already accumulated in vascular health, causing this endothelium to be less affected in exacerbation of the disease, while patients who walk a minor distance may have a worse vascular prognosis. In general, physical exercise has been shown to improve exercise capacity and arterial function in particular endothelium-dependent vasodilation^44^, which seems to be an important key to upgrade vascular health and CV events prevention at AECOPD.

The increased inflammatory state associated with AECOPD may even further accelerate cardiovascular diseases and place patients at an increased risk of death due to cardiac events at this moment^45^. Our findings on endothelial function values are close to those from Marchetti et al.^17^, that demonstrated FMD values of 2.8%, while in present study the values were 4.0%. In the study of Ozben et al.^46^, patients presented about 6% of FMD, though the exacerbation was not severe. The exercise is also responsible for decreasing levels of inflammatory markers such as cytokines and C-reactive protein^44^. A study described for the first time the association between airway inflammation and endothelial dysfunction related to NO activity in patients with COPD^47^. They demonstrated that endothelial NO function was compromised by suppressing airway inflammation. We emphasize that this study may further reinforce our findings that a lower exercise capacity in hospitalization would be related to a worse vascular outcome of patients with acute exacerbation of COPD.

A recent review showed that worsening of muscle strength, physical activity, and exercise performance during hospitalization due to exacerbation may be a marker for further worsening in the future. Given the evidence, the AECOPD period represents a crucial time to explore interventions that can reverse these processes and reduce the morbidity associated with hospitalization for AECOPD^48^.

Although our results have demonstrated a positive association between exercise capacity and endothelial function, in which 6MWD accounting for 15% of the FMD variance in severe AECOPD patients, a causal relationship cannot be established. However, our findings should encourage researchers to elucidate the effect of rehabilitative strategies to enhance exercise capacity have on endothelial function, CV risk and clinically relevant outcomes to optimally manage this high-risk population. In addition, the prognostic value of impaired exercise capacity regarding endothelial function, cardiovascular health and functional status in hospitalized patients with AECOPD also deserve to be explored.

### Limitation of the study

The present study has some limitations. For instance, its cross-sectional design, which does not allow the establishment of a causal relationship. Furthermore, only one method to assess endothelial function was used and endothelial-independent dilation was not measured.

## Conclusion

The findings of this study confirmed our hypothesis that exercise capacity is associated with endothelial function in patients with chronic obstructive pulmonary disease and severe AECOPD. Endothelial function was found to be increasing with increasing walked distance. These results highlight a possible and valuable potential of rehabilitation strategies focusing on improving exercise capacity translate into vascular and CV risk benefits. However, further research is required to provide evidence of effectiveness of this rehabilitation programs.

## Data Availability

The datasets used and/or analysed during the current study are available from the corresponding author on reasonable request.
